# Hybrid of Graphene based on quaternary Cu_2_ZnNiSe_4_ –WO_3_ Nanorods for Counter Electrode in Dye-sensitized Solar Cell Application

**DOI:** 10.1038/s41598-020-61363-x

**Published:** 2020-03-16

**Authors:** Won Chun Oh, Kwang Youn Cho, Chong Hun Jung, Yonrapach Areerob

**Affiliations:** 1 College of Materials Science and Engineering, Anhui University of Science & Technology, Huainan, 232001 P.R. China; 2 Department of Advanced Materials Science & Engineering, Hanseo University, Seosan-si, Chungcheongnam-do 31962 South Korea; 3 Korea Institute of Ceramic Engineering and Technology, Soho-ro, Jinju-Si, Gyeongsangnam-do South Korea; 4 Decontamination & Decommisioning Research Division, Korea Atomic Energy Research Institute, P.O. Box 105, Yuseong-gu, Daejeon 305-600 South Korea

**Keywords:** Devices for energy harvesting, Electronic properties and devices

## Abstract

A novel nanohybrid of graphene-based Cu_2_ZnNiSe_4_ with WO_3_ nanorods (G-CZNS@W) was successfully synthesized via a simple hydrothermal method to use as a counter electrode (CE) for dye-sensitized solar cells (DSSCs). The characterization technique confirmed the structural and morphologies of the G-CZNS@W nanohybrid, which could show rapid electrons transfer pathway through the WO_3_ nanorods. Moreover, the as-fabricated G-CZNS@W nanohybrid exhibited synergetic effect between G-CZNS and a WO_3_ nanorod, which could affect the electrocatalytic activity towards triiodide reaction. The nanohybrid exhibits an excellent photovoltaic performance of 12.16%, which is higher than that of the standard Pt electrode under the same conditions. The G-CZNS@W nanohybrid material as CE thus offers a promising low-cost Pt-free counter electrode for DSSC.

## Introduction

Extracting energy from fossil fuels is the major cause of environmental pollution. Solar energy, a source of renewable energy, could be considered as an alternative source of energy.

Dye-sensitized solar cells (DSSCs) are the most promising renewable energy devices, as was reported by Michael Grätzel in 1991^1^. These DSSCs devices have been introduced into the market to convert the renewable incident solar radiation into electricity with high power conversion efficiency, low-cost fabrication, and in an environmentally benign manner^2^. Generally, DSSCs consist of (1) the working electrode, which is coated by a thin, mesoporous layer of a semiconductor, usually TiO_2_, on whose surface a monolayer of dye molecules is adsorbed; and (2) the counter electrode, which is coated with a thin catalyzer layer, usually Pt electrode. The space between the two electrodes is filled with an electrolyte containing a redox couple (often I^−^/I_3_^−^)^3,4^.

Major research efforts have been undertaken to find alternatives to the traditional Pt CE. Because of its high cost and low resources, a better alternative to Pt is required for commercial applications. Some of the counter electrodes with high efficiency reported so far have included carbonaceous materials, such as carbon^5^, graphene^6^, and N-doped carbon^7^; polymers, such as Polyaniline nanotube^8^, PEDOT:PSS/halloysite^9^, and poly α-naphtylamine^10^; metal sulphides, such as PbS^11^, FeS_2_^12^, and CoS_2_^13^; metal oxides, such as CoFe_2_O4^14^, MnO_2_^15^, and WO_3_^16^; and quaternary material, such as Cu_2_ZnSnS_4_^17^, and La_1−x_Ca_x_MnO_3_^18^.

Among these materials, quaternary composite material has drawn much interest, due to its unique hybrid structure with low bandgap structure, adaptability of photoelectrochemical performance, and long-term stability; and the high surface area of quaternary material provides more active sites for receiving electrons from external circuits, while reducing the triiodide ion back to an iodide ion through an efficient charge transfer process^19^. Moreover, various research groups have previously explored the capability of Metal oxide as counter electrode, because of its excellent electrical conductivity, good catalytic activity, and good stability^20,21^. However, the preparation of these CE compounds requires either more complicated reaction routes, or a long reaction cycle for increased efficiency in DSSCs.

Graphene two-dimensional crystal with a hexagonal lattice has been extensively combined with metal oxide materials. It demonstrates unique properties, such as an excellent electron transport pathway. Rahman *et al*. reported cobalt sulfide on graphene nanosheets, and used it as a counter electrode with a power conversion efficiency of 5.48%^22^. It is evident that it provides a lower yield than the traditional Pt electrode. In addition, the synergistic effect is an effect when two or more materials are combined, which materials can provide bridge structure in the composite. The bridge structure affects the electron transfer or recombination of materials, which means the resistance of the cell. Therefore, the synergistic effect can decrease the resistance properties, which can increase the power conversion efficiency^23^.

Hence, the Cu_2_ZnNiSe_4_ (CZNS) has high electrocatalytic activity, good electrochemical stability, and a large surface area for rapid interface reactions^24^. To the best of our knowledge, there have been no reports on the application of CZNS in DSSC. Herein, Hybrid Graphene – Cu_2_ZnNiSe_4_ – WO_3_ nanorods (G-CZNS@W) were synthesized by hydrothermal methods. Structurally, the WO_3_ nanorod increases the electrical conductivity of the CZNS material by the bridging, while the graphene nanoparticles offer a large surface area for the electrochemical reactions. This G-CZNS@W nanocomposite showed a low charge-transfer resistance, thus making it a promising electrode material for DSSC.

## Experimental setup

### Chemical, reagents, and characterization

All chemicals were purchased from Merck, Korea (KR), and were used without any additional purification. Fluorine-doped tin oxide (FTO) conducting glass slides (7 V/cm^2^, Sigma-Aldrich), TiO_2_ powder (P25, Degussa AG,) Sodium hydroxide pellets (NaOH, GR grade), hydrochloric acid (HCl 30% in aqueous solution, GR grade), and ethanol (GR grade) were purchased from Sigma-Aldrich. We examined the composition of all sample powders by X-ray diffraction (XRD, Cu K radiation, Smart Lab 3 kW, Rigaku, Japan). We used Lambda 950 UV-vis-NIR spectrophotometry to evaluate the optical transparencies. We evaluated the photovoltaic performance of the fabricated DSSCs using a calibrated A.M 1.5 solar simulator (Newport) with a light intensity of 100 mW/cm^2^ and a computer-controlled digital source meter (Keithley, Model 2420).

### Synthesis of graphene oxide

We synthesized graphene oxide (GO) using a modified Hummer’s method^25^. Briefly, 5 g of Graphite power were added to 50 mL of H_2_SO_4_ solution under continuous stirring for 30 min at 0 °C, followed by the addition of 30 g KMnO_4_. Then, diluted H_2_O_2_ was added to the above solution, which was stirred for 1 h, and the solution temperature kept at 100 °C. After cooling, the final solution was repeatedly washed with 1 M HCl, followed by de-ionized (DI) water. After drying at 60 °C overnight, GO was re-dispersed in DI water, subjected to sonication for 10 min, and centrifuged at 4,000 rpm to remove unexfoliated GO.

### Synthesis of Cu_2_ZnNiSe_4_ (CZNS) and WO_3_ nanorods

We synthesized CZNS nanoparticles by solid-state reaction, as reported in our earlier work^26^. In brief, the appropriate molar ratios of elemental precursors (Cu:Zn:Ni:Se) were taken in a 30 mL polypropylene bottle^26^. This 10 mL of ethanol was added as a solvent, and kept for ultra-sonication for 30 min. After that, we added an appropriate amount of zirconia balls of 3 mm into the bottle, and it was kept for wet pot milling for 24 h at 90 rpm, to get a homogeneous mixture. After pot milling, the homogenized precursor solution was kept at 400 °C for 2 h in inert atmosphere. After synthesis, the powders were ground for further characterization.

We synthesized the WO_3_ nanorods by the hydrothermal method. The experimental details were as follows. We dissolved 0.05 M Na_2_WO_4_ × 2H_2_O in 50 mL of deionized water under constant magnetic stirring, to form a clear solution. Subsequently, we added 1 M HCl solution dropwise, to reach a pH of ~1.0. Then, the solution was transferred into a 100 mL Teflon-lined stainless-steel autoclave, which was heated at 180 °C in an electric oven for 12 h, and then naturally cooled to room temperature (RT).

### Synthesis of Graphene–Cu_2_ZnNiSe_4_–WO_3_ nanorods (G-CZNS@W)

An aqueous solution consisting of 0.5 g GO, 0.25 g CZNS, and 0.25 g WO_3_ at stoichiometric ratios was mixed. We adjusted the total volume of the reagent solution to 50 mL by adding deionized water. After vigorous agitation for 10 min, 50 mL of the reactant was transferred into a Teflon-lined autoclave, and kept at 150 °C for 10 h. After cooling, the precipitate was washed several times by DI water and ethanol. Finally, the precipitate was dried at 80 °C for 12 h. The schematic diagram (Fig. S.1) shows the synthetic process.

### Fabrication of DSSCs

Firstly, 1 g of TiO_2_ powder was mixed with 5 mL of ethanol, and crushed by mortar and pestle for 5 min. Next, the photoanode TiO_2_ thin film was coated onto an FTO glass substrate by a doctor blade method^2^. The TiO_2_ thin films were then calcined at 450 °C for 30 min. After cooling to RT, the resultant nanocrystal films were further sensitized by immersion into a 0.50 mM ethanol solution of N719 dye for 1 day. Then the TiO_2_ films were washed with DI water for five times.

For counter electrode, we coated the CZNS, WO_3_ and G-CZNS@W paste on FTO substrates via the same method. The G-CZNS@W films were sintered at 500 °C for 30 min. The heat-treated G-CZNS@W films were then ready to be used as CE in DSSC. Then, the cells were coupled using 25 µm thick Surlyn (Solaronix) as a spacer between the photoelectrode and CE. We injected the electrolyte into the hole drilled in the counter electrode, using vacuum suction to ensure complete filling. The active area of the device was set to 0.25 cm^2^ during the *J–V* measurements.

## Results and Discussion

### XRD analysis

**Figure 1:**
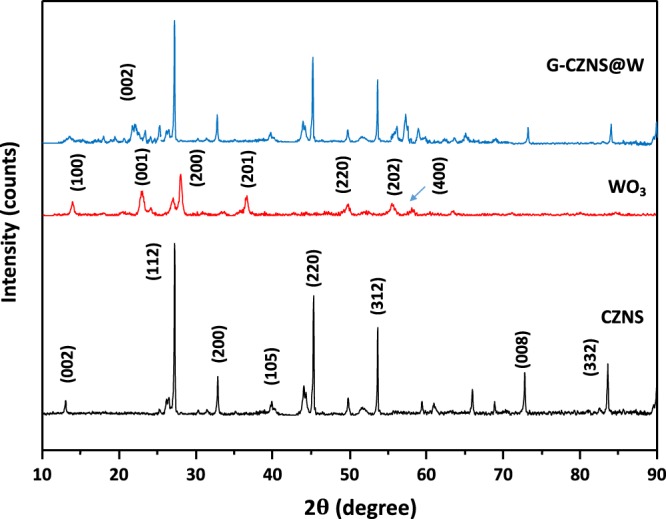
XRD patterns of the CZNS, WO_3_, and G-CZNS.

Figure 1 shows the X-ray diffraction (XRD) pattern of the synthesized CZNS, WO_3_, and G-CZNS@W. The three dominant peaks (112), (220), and (312) are attributed to the CZNS kesterite phase, indexed based on the standard JCPDS data (JCPDS card No. 26-0575)^27^. For the WO_3_ nanorod, it was evident that all the sharp diffraction peaks could be classified as the monoclinic phase of WO_3_, and the results matched well with the standard JCPDS data (card No. 83-0951)^28^. Moreover, we observed the graphene peak (002) at 2θ of ~26°.

### SEM-based EDS and TEM analysis

**Figure 2:**
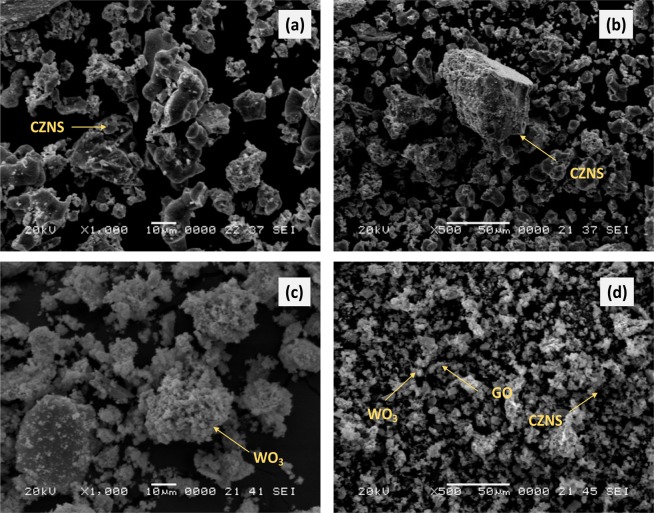
SEM images of (**a,b**) CZNS, (**c**) WO_3._ and (**d**) G-CZNS@W.

**Figure 3:**
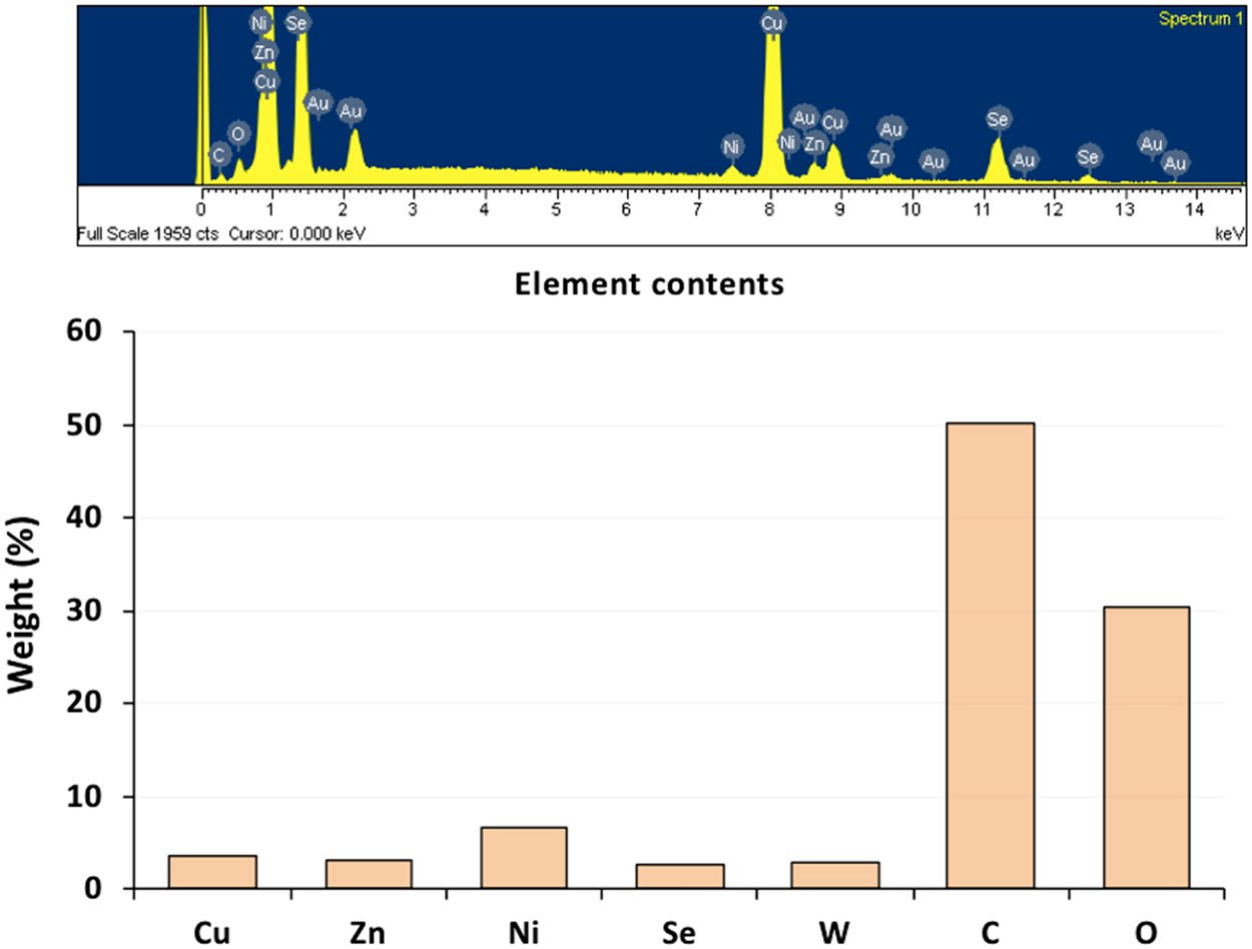
EDX spectra of G-CZNS@W.

We examined the morphologies and microstructures of the CZNS, WO_3_, and G-CZNS@W by scanning electron microscopy (SEM) and transmission electron microscopy (TEM). Figure 2(a,b) showed the absence of CZNS with irregular topography tended to aggregate together, and a particle size of ~(50–100) nm. Figure 2(c) shows the heterogeneous structure with numerous clusters of particles in irregular sphere-like structures of WO_3_. In addition, Fig. 2(d) shows the morphology of the CZNS@W nanocomposite consisting of graphene sheets. The graphene sheets were of approximately 20 nm thickness, with WO_3_ and CZNS spread on the top of the surface from each other, and in random array. Figure 3 shows the EDS analysis that confirms the elements of Cu, Zn, Ni, Se, W, C, and O were deposited on the G-CZNS@W nanocomposites.

**Figure 4:**
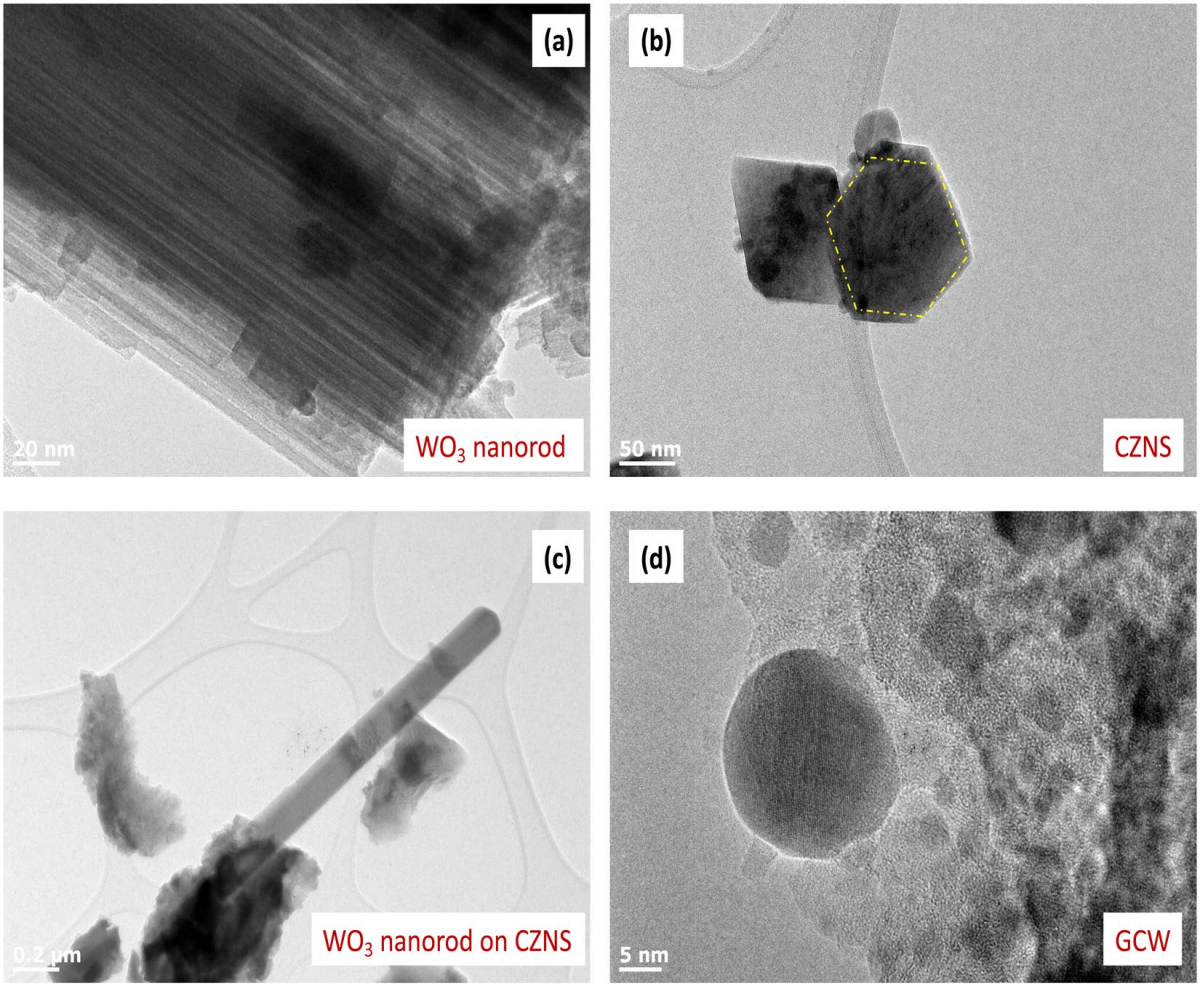
TEM images of (**a**) WO_3_ nanorod, (**b**) CZNS, (**c**) G-CZNS, and (**d**) G-CZNS@W.

Furthermore, Fig. 4(a) shows the TEM image of the WO_3_ nanorods. The surface of the WO_3_ composite can be clearly seen to be composed of tiny nanorods, which facilitate easy diffusion of electrolyte ions, and improve the electron transport pathway. Moreover, the WO_3_ nanorods were continuous, and showed a smooth surface morphology. Figure 4(b) provides a plane view of CZNS that shows a granular structure of about 50 nm, which provided more active sites and high surface area for triiodide reduction. In contrast, the CZNS functionalized on the WO_3_ nanorods showed very rough surfaces, because of the presence of CZNS nanoparticles in their surfaces, as shown in Fig. 4(c). Figure 4(d) shows the surface morphology of the CZNS@W. The CZNS particles and WO_3_ nanorods can be seen to be homogeneously coated by GO sheets, which can confirm the strong interaction effect between GO and CZNS@W, and which provides excellent electric performance^29^.

### X-ray photoelectron analysis

**Figure 5:**
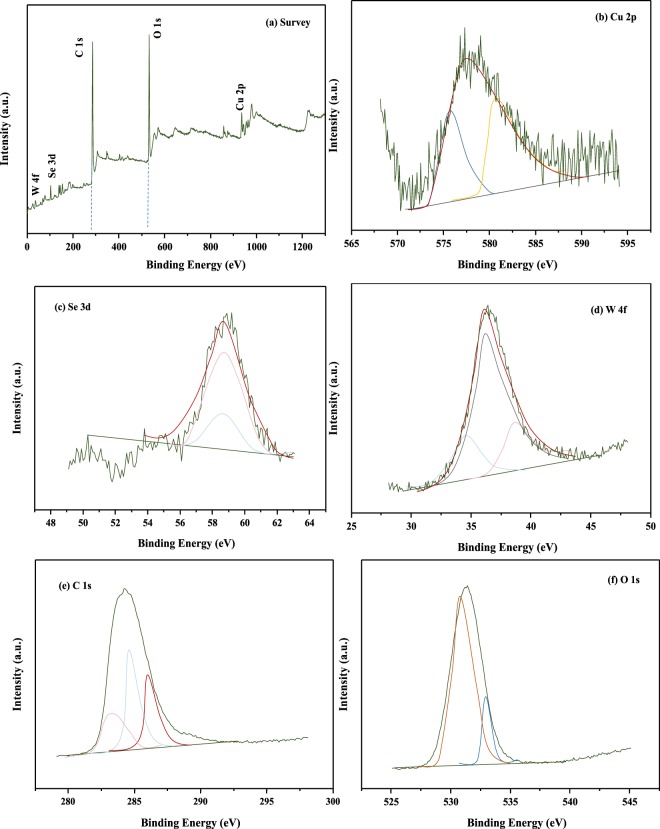
(**a**) Survey XPS spectrum; (**b**) XPS spectra of Cu 2p, (**c**) Se 3d, (**d)** W 4f, (**e**) C 1 s, and (**f**) O 1 s.

To further investigate the bonding configurations and confirm the elemental compositions of the G-CZNS@W nanohybrid, we carefully measured the X-ray photoelectron spectra (XPS). These show the existence of G-CZNS@W that includes the spectra of Cu, Se, W, C, and O, as well as the corresponding chemical bonds, as confirmed by Fig. 5(a). Figure 5(b) shows the binding energies at 577.25 eV that belong to the Cu 2p_1/2_ in the CZNS nanohybrid. In particular, in Fig. 5(c), the peak at 59.4 eV for the Se 3d spectrum is consistent with Se 3d_5/2_ via fitting the peak of the specific peak position and oxidation state of the elements. In the WO_3_ nanorod, the high-resolution spectra of W 4 f (Fig. 5(d)) show one peak with a binding energy of 37.72 eV, which can be ascribed to W4f_5/2_. Similarly, the binding energies of C and O also match the standard spectra well, as demonstrated by Fig. 5(e,f) ^30–32^. Therefore, the graphene sheets are composed of CZNS@W on the surface, which is a good chemical state for the G-CZNS@W CE.

### Raman spectra analysis

**Figure 6:**
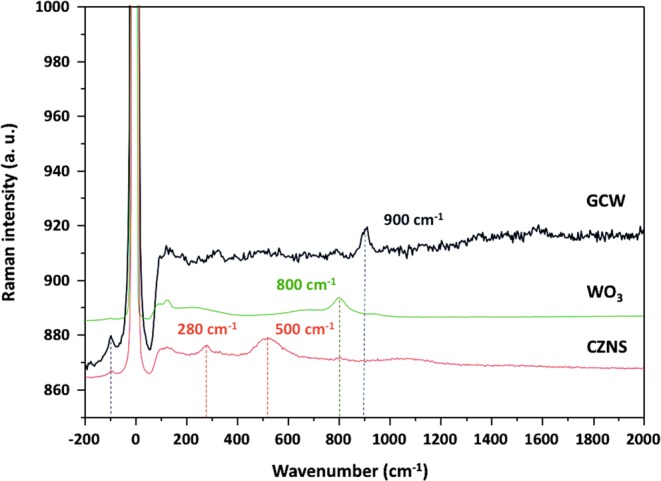
Raman spectra of the as-synthesized CZNS, WO_3_, and G-CZNS@W.

We used Raman spectroscopy to characterize the structural information of the CZNS, WO_3_, and G-CZNS@W materials (Fig. 6). From the CZNS spectra, two distinct peaks at (280 and 500) cm^−1^ confirm the E_2g_ peak of the metal hybrid in the composite. The Raman spectra of WO_3_ nanorod located at (135 and 270) cm^−1^ can be attributed to the antisymmetric stretching vibration of (W–O–W) and bending mode of the (W–O–W) bonds^32^. Moreover, two distinct peaks of the D and G bands at (1,348 and 1,590) cm^−1^, respectively, were observed in the G-CZNS@W. The D band is related to the edges or disordered layers, while the G band corresponds to the E_2g_ mode of sp^2^ carbon atom^33^. We calculated the intensity ratios of the D to G bands (I_D_/I_G_) to be 1.01 for G-CZNS@W. The higher I_D_/I_G_ ratio of G-CZNS@W suggests that more defective sites were introduced, which can increase the electrocatalytic activity of the CE.

### N_2_ adsorption and desorption isotherms

**Figure 7:**
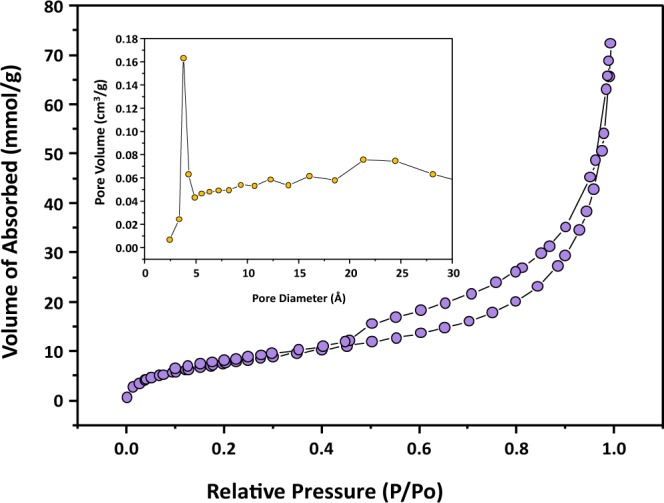
The N_2_ adsorption-desorption isotherms for G-CZNS@W samples and (inset) BJH pore-size distribution plot for the G-CZNS@W sample.

Figure 7 shows the nitrogen adsorption–desorption measurements by which we characterized the mesoporous structure and surface area of the G-CZNS@W thin films. The figure shows typical type IV isotherms with the H_3_ hysteresis loop, which confirm the mesoporous structure of the samples. Moreover, the pore-size distribution plot for the G-CZNS@W sample (inset) is in the range (2–5) nm, which suggests the mesoporous structure of the G-CZNS@W samples. The calculated specific surface area for G-CZNS@W was 59.6 m^2^ g^−1^. These results suggest that the high surface area for the G-CZNS@W samples helps improve the super-capacitive performance of the electrode, because it provides large electrochemically active sites, and numerous channels for ion transport during electrochemical reactions^34^.

### UV-DRS analysis

**Figure 8:**
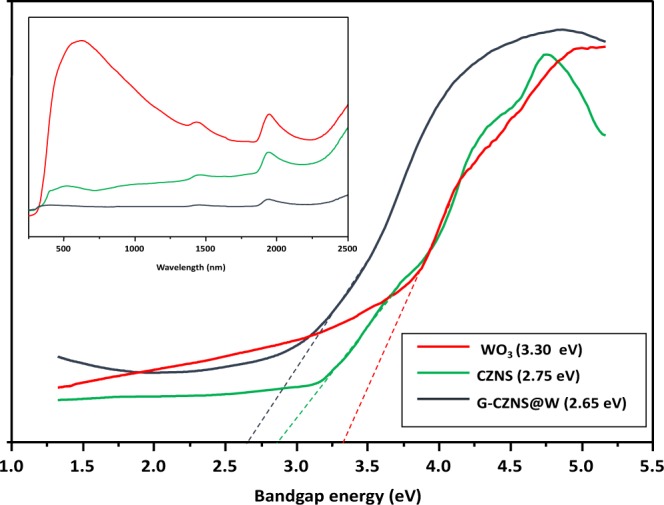
Band-gap energy and Uv-Vis spectra (inset) of WO_3_, CZNS, and G-CZNS@W.

Figure 8 shows the bandgap energies of the CZNS, WO_3_, and G-CZNS@W composites, which it was necessary to investigate. We found that all of the samples showed absorption edges in the range (350–700) nm. Additionally, Fig. 8 shows the plot of α^2^ versus *hν* of the CZNS, WO_3_, and G-CZNS@W nanocomposites, in which *a* and *hν* represent the parameter of optical absorption and the energy of the incident photon, respectively. According to these plots, the energy gaps (*E*g) of CZNS, WO_3_, and G-CZNS@W are (2.75, 3.30, and 2.65) eV, respectively. According to the results, the G-CZNS@W composite has a lower bandgap energy due to the CZNS, and the WO_3_ can promote the electrons from valance to conduction band, as well as produce a large number of photo-generated electron–hole pairs in the surface of the graphene sheets, which benefits the electron transfer ability^35^.

### The photocurrent density-voltage *(J–V)* curves

**Figure 9:**
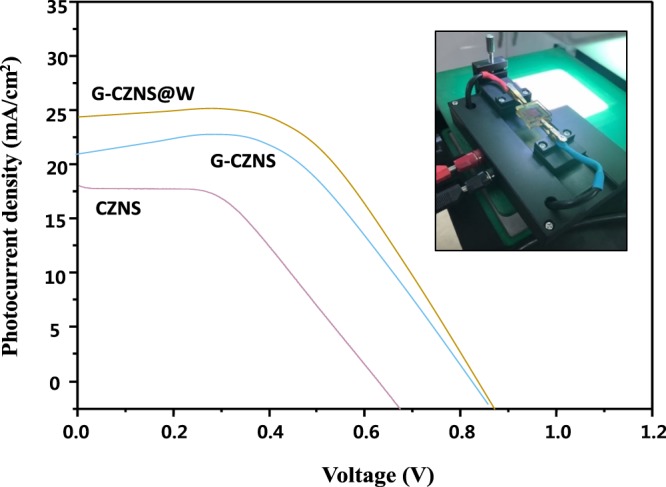
I-V typical curves of CZNS, G-CZNS, and G-CZNS@W.

The electrical and optoelectronic properties, including the photoelectric conversion performance of the CZNS, G-CZNS, and G-CZNS@W CE samples, were measured by the photocurrent density–voltage (*J–V*) curves, as shown in Fig. 9 and Table 1. The DSSC fabricated with G-CZNS@W CE exhibits the highest power conversion efficiency (PCE) of 12.16%, which is higher than those of CZNS (3.88%), G-CZNS (8.75%), and the traditional Pt CE (4.07%)^25^.

**Table 1:** DSSCs Performance Using CZNS, G-CZNS, and G-CZNS@W.

CEs	Voc (V)	Jsc (mA cm^−2^)	FF (%)	PCE (%)	Ref.
Pt	0.66	7.80	70.01	4.07	^25^
CZNS	0.68	17.50	32.60	3.88	This work
G-CZNS	0.86	21.21	47.97	8.75	This work
G-CZNS@W	0.88	24.70	55.95	12.16	This work

It can be seen that the *J*_sc_ was increased, which can improve the electrocatalytic activity and charge transfer of the G-CZNS@W nanocomposite. The G-CZNS@W has more electrocatalytically active sites for triiodide reduction with faster electron-transfer kinetics at the counter electrode/electrolyte interface, because of the interconnection between uniformly distributed electro-catalytic CZNS nanoparticles, and the intrinsic electrical conductivity of graphene^36^. Moreover, the WO_3_ nanorod builds up an efficient three-dimensional catalytic network, and maximizes the fraction of exposed active edge sites for the reduction of I_3_^−^, which can increase the efficiency in DSSC.

### Electrochemical impedance spectroscopy (EIS)

**Figure 10:**
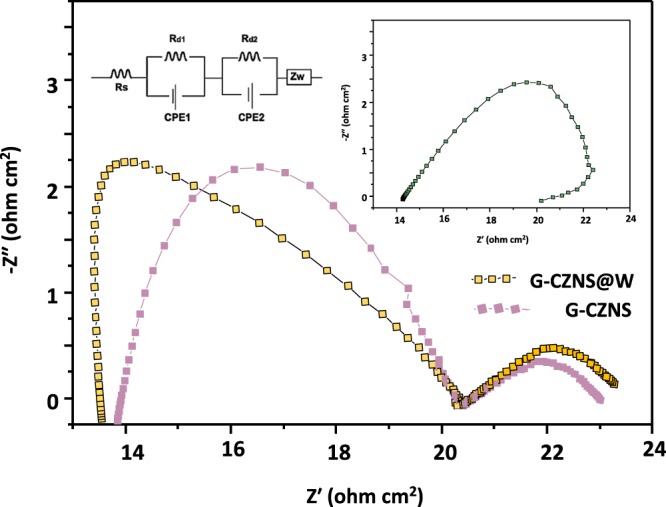
Nyquist plots of CZNS, G-CZNS, and G-CZNS@W and equivalent circuit (inset).

**Figure 11:**
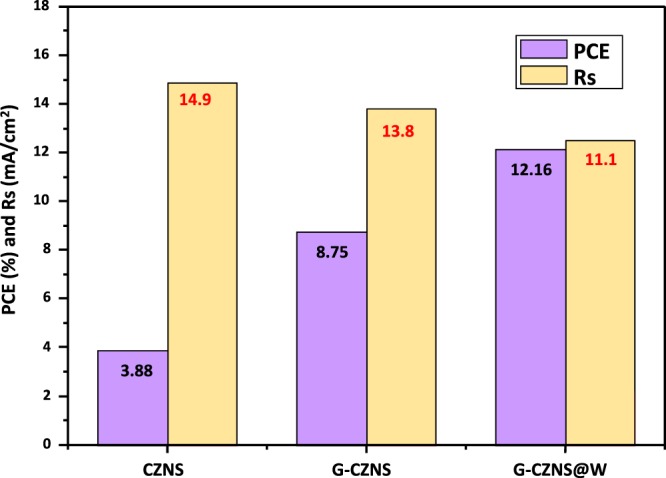
Power-conversion efficiency (PCE) and series of resistances (*R*s) of CZNS, G-CZNS, and G-CZNS@W.

Electrochemical impedance spectroscopy (EIS) on symmetrical cells consisting of two identical CEs was conducted to investigate the electrochemical reactions occurring at the electrode/electrolyte interface. Figure 10 and Table 2 present the obtained Nyquist plots of the three electrodes. The high-frequency intercept on the real axis is attributed to a series of resistances (Rs) of the cell components, while the left semicircle in the high-frequency range is assigned to the charge–transfer resistance (Rct) at the electrode/electrolyte interface. The Rs value of 11.10 Ω·cm^2^ for G-CZNS@W CE is smaller than that for G-CZNS (13.8 Ω·cm^2^), which can be attributed to the strong interaction between the G-CZNS and WO_3_ layers. Moreover, the Rct of G-CZNS@W CE is 2.52 Ω·cm^2^, which is much smaller than that of G-CZNS (2.91 Ω·cm^2^), indicating that the G-CZNS@W electrode is more efficient in terms of catalytic reduction of I_3_^−^ at the CE/electrolyte interface. The improvement of the catalytic activity of the G-CZNS@W CE can be ascribed to the abundant active edge sites on the high-curvature surface of the WO_3_ nanorod^37–39^. Moreover, Fig. 11 shows the comparison of the power-conversion efficiency (PCE) and a series of resistances (Rs) of CZNS, G-CZNS, and G-CZNS@W.

**Table 2:** Corresponding Parameters of the Nyquist Plots of CZNS, G-CZNS, and G-CZNS@W.

CEs	Rct (Ω)	Rs (Ω)	Ref.
Pt	1.79	5.62	^25^
CZNS	6.21	14.90	This work
G-CZNS	2.91	13.80	This work
G-CZNS@W	2.52	11.10	This work

Graphical Abstract.

Schematic diagram of Graphene-Cu_2_ZnNiSe_4_-WO_3_ nanorod preparation.

## Conclusion

In this work, we successfully synthesized G-CZNS@W via a simple hydrothermal method, and used it as a CE in DSSC. The G-CZNS@W exhibited higher electrocatalytic activity toward the triiodide reaction, and rapid charge-transferability. The synergy between G-CZNS@W and graphene improved the performance of the DSSC. Moreover, it showed the larger active surface area and strong interaction of the WO_3_ with the G-CZNS. The DSSC fabricated using G-CZNS@W CE showed a photo-conversion efficiency of 12.16%, which is higher than that of the DSSCs fabricated using Pt (4.07%). These results demonstrate that the G-CZNS@W nanohybrid could be used as a platinum-free counter electrode for high-performance DSSCs.
